# Sleeve gastrectomy attenuated diabetes-related cognitive decline in diabetic rats

**DOI:** 10.3389/fendo.2022.1015819

**Published:** 2022-11-03

**Authors:** Huanxin Ding, Chuxuan Liu, Shuo Zhang, Bingjun Li, Qian Xu, Bowen Shi, Songhan Li, Shuohui Dong, Xiaomin Ma, Yun Zhang, Mingwei Zhong, Guangyong Zhang

**Affiliations:** ^1^ Department of General Surgery, Shandong Provincial Qianfoshan Hospital, Cheeloo College of Medicine, Shandong University, Jinan, China; ^2^ Medical Research Center, Shandong Provincial Qianfoshan Hospital, Cheeloo College of Medicine, Shandong University, Jinan, China; ^3^ Department of General Surgery, The First Affiliated Hospital of Shandong First Medical University, Jinan, Shandong, China; ^4^ Department of Breast Disease, Peking University People’s Hospital, Beijing, China

**Keywords:** diabetes-related cognitive decline, sleeve gastrectomy, PI3K, tau, neuronal apoptosis

## Abstract

**Objective:**

To investigate the effects of sleeve gastrectomy (SG) on diabetes-related cognitive decline (DCD) in rats with diabetic mellitus (DM).

**Methods and methods:**

Forty Wistar rats were randomly divided into control (CON) group (n=10), diabetes mellitus (DM) group (n=10), sham operation (SHAM) group (n=10) and SG group (n=10). DM model was established by high-fat diet (HFD) combined with intraperitoneal injection of streptozocin (STZ). Behavioral evaluation was given using Morris water maze test and Y-maze. In addition, PET-CT, TUNEL assay, histological analysis, transmission electron microscopy (TEM), immunohistochemistry (IHC) and Western blot analysis were used to evaluate the alleviating effects and potential mechanisms of SG on DCD in DM rats.

**Results:**

Compared with the sham group, SG induced significant improvement in the metabolic indices such as blood glucose and body weight. Besides, it could attenuate the insulin resistance compared with SHAM group. In addition, SG could improve the cognitive function of DM rats, which were featured by significant decrease in the escape latency (P<0.05), and significant increase in the time in target quadrant and platform crossings (P<0.05) compared with the SHAM group. SG induced significant elevation in the spontaneous alternation compared with SHAM group (P<0.05). Moreover, SG could improve the arrangement and biosynthesis of hippocampus neuron. Moreover, SG triggered the inhibition of apoptosis of hippocampus neurons, and Western blot analysis showed SG induced significant increase in the ratios of Bcl-2/Bax and Caspase3/cleaved Caspase 3. TEM demonstrated SG could significantly improve the microstructure of hippocampus neurons compared with the SHAM group. Western blot and IHC confirmed the significant decrease in the phosphorylation of tau at Ser404 and Ser396 sites in the SG group. Furthermore, SG activated the PI3K signaling pathway by elevating the phosphorylation of PI3K and Akt and GSK3β compared with the SHAM group.

**Conclusion:**

SG attenuated the DCD in DM rats, which may be related to the activation of PI3K signaling pathway.

## Introduction

Diabetes mellitus (DM), with a sharp increase in the prevalence in the last three decades, is considered the ninth leading cause of death worldwide (1). Patients with DM usually show involvement of the nervous system, resulting in cognitive decline (2, 3). As a major complication of DM, the diabetes-related cognitive decline (DCD) is characterized by abnormalities in learning, memory, attention and speed of information processing (4). To date, impairment of hippocampus has been closely associated with the learning and memory loss, while in animals with DCD, the main pathological features of the hippocampus are neurogenic fibrillary tangles due to tau hyperphosphorylation and neuronal apoptosis (5).

Bariatric surgery, initially utilized for treating morbid obesity, is later reported to show a moderate effect on DM and its complications (6, 7). Bariatric surgery can ameliorate hyperglycemia (8) and reduce the body weight (9). In addition, patients underwent such type of surgery showed significant improvement in the cognitive function (10).

PI3K/Akt signaling pathway plays a key role in the pathogenesis of DCD (11, 12), which mediates biological growth and crucial cellular metabolic processes, such as glucose homeostasis, lipid metabolism, protein synthesis and cell proliferation and survival (13). In diabetic rats or glucose-induced hippocampal neuronal impairments, there was decrease in the phosphorylation of AKT, resulting in a decrease in the phosphorylation level of glycogen synthase kinase 3β (GSK3β) (14, 15), serving as a key enzyme that inhibits glycogen synthesis and one of the key kinases for tau phosphorylation (16). Physiologically, activation of the PI3K insulin signaling pathway could inhibit the hippocampal neuronal apoptosis. In addition, it could ensure that tau phosphorylation is maintained at a normal level by inactivating GSK3β (17).

Sleeve gastrectomy (SG) serving as one of the most popular bariatric surgeries for obesity has been reported to improve the cognitive function among the obesity patients (18, 19). However, little is known about the exact mechanisms for this process. In this study, a DM rat model was established in order to investigate how SG improved the cognitive function, and at the same time, we determined the activity of PI3K/AKT signaling pathway, with an aim to illustrate its roles in this process.

## Materials and methods

### Animals

Forty male Wistar rats (90–110g; 6-week-old), purchased from Vital River Laboratory Animal Technology (Beijing, China), were housed in the animal laboratory of Shandong Provincial Qianfoshan Hospital of Shandong University, under specific pathogen-free housing conditions at 20–26°C in a humidity of 50–60%. All animals were fed on a standard diet containing 15% of fat for 1 week for acclimatization. This animal study was approved by the Institutional Animal Care and Use Committee of Shandong Qianfoshan Hospital, Shandong University.

### Grouping

**Figure 1:**
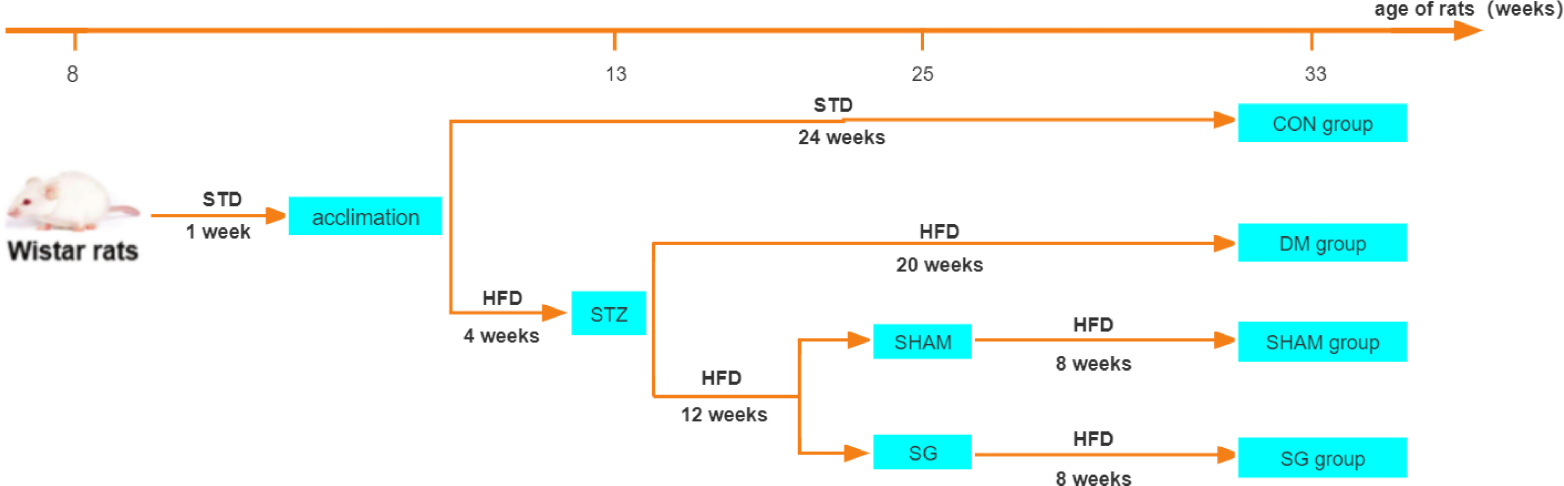
Flowchart of animal experiments. N = 10 for each group. STD, standard diet; HFD, high-fat diet; STZ, streptozotocin; CON, control group; DM, diabetes mellitus; SHAM, sham operation; SG, sleeve gastrectomy.

The animals were randomly divided into the following four groups: (i) control (CON) group (n=10), rats subject to a standard diet; (ii) DM group (n=10), rats subject to DM induction; (iii) sham operation (SHAM) group (n = 10), and (iv) SG group (n=10). One week before the surgical intervention in the SG and SHAM groups, Y-maze test was performed to confirm the differences in cognitive ability among the groups (**Figure S1**). Animals in the SG group were subject to SG after a 12 hrs fast. Animals in the SHAM group were subject to DM induction, followed by operations that were the same as the above surgery before occlusion of gastric blood vessels. There were no interventions in the SHAM group except exposure of abdominal organs such as the stomach, small intestine, and liver. The flowchart of the study was shown in **Figure 1**.

### DM induction

Animals were given a 4-week high fiber diet (HFD, 40% fat; Xietong Pharmaceutical Bio-engineering, Nanjing, China), followed by a 12 hrs fast. Then the rats received a single intraperitoneal injection of streptozocin (STZ, 35 mg/kg, Sigma-Aldrich, St. Louis, MO, US). Three days after STZ injection, random blood glucose was measured 3 times consecutively, and a glucose level of ≥ 16.7 mmol/L was considered a successful model (20).

### SG procedures

The procedures of SG were performed as previously reported (21). All rats were fasted for 12 hrs before surgery and then anesthetized by continuous inhalation of 2% isoflurane gas using a mask. First, a 4 cm incision was made in the middle of the upper abdomen. Then, the stomach was separated until a clear distinguish of the gastric blood vessels. The blood vessels of the fundus and the greater curvature of the stomach were ligated with a 7-0 thread (Chenghe Microsurgical Instruments, Ningbo, China), and then cut off after ligation. Subsequently, an incision of about 0.5 cm parallel to the greater curvature of the stomach was made in the fundus to remove the gastric contents. The fundus and most of the gastric body on the greater curvature of the stomach were removed, and the remaining part was sutured with 5-0 sutures (Cheng-He Microsurgical Instruments Factory, Ningbo, China). No active bleeding was confirmed and the anatomical position of the abdominal organs was restored. Finally, close the abdominal wall layer by layer with 3-0 sutures (Chenghe Microsurgical Instruments, Ningbo, China).

### Body weight and food intake measurement

For the animals in each group, we measured the baseline body weight and food intake. Then the body weight and food intake were measured at 2, 4, 6, and 8 weeks afterwards.

### Homeostasis model assessment of insulin resistance

Upon fasting for 12 hrs, the blood samples were collected from the tail vein after anesthesia, and centrifuged at 3,000 rpm for 8 min. Fasting blood glucose (FBG) was measured using a blood glucose meter (One Touch Ultra, Johnson & Johnson, CA, USA). Then serum insulin was detected with EZRMI-13K kit (One Touch Ultra, Johnson & Johnson, CA, USA). Finally, the Homeostasis Model Assessment of Insulin Resistance (HOMA-IR) was calculated to assess the degree of insulin resistance, using the following formula: HOMA-IR=fasting serum insulin (mIU/L)×FBG (mmol/L)/22.5 (22).

### Oral glucose tolerance test and insulin tolerance Test

For the OGTT, glucose (1 g/kg) was given to each rat *via* intragastrical administration. Then the blood glucose was measured at 0, 10, 30, 60, and 120 min, respectively. About 24 hrs after OGTT, ITT was performed after a 12-hour fast. The rats were intraperitoneally injected with insulin (0.5 IU/kg, Tonghua Dongbao Pharmacy. Gansu, China), and then the blood glucose was measured at 0, 10, 30, 60, and 120 min, respectively. Finally, the area under the curve (AUC) of OGTT and ITT was calculated with the trapezoidal method according to the previous description (23, 24).

### Morris water maze test

MWM test was used to measure hippocampus-dependent cognitive function as previously described (25). All behavioral tests were performed during the active period of the photoperiod. A video analysis system (Calvin Biotech, Nanjing, China) was utilized to record the swimming patterns of each rat. The pool was filled with water at a constant temperature of 20°C. An escape platform with a diameter of 4.5 cm was placed in the swimming pool. The top of the platform was approximately 1.5 cm below the water surface.

The 5-day hidden platform test was used to detect the learning ability of rats. Rats were trained for 5 days. Animals failed to find the platform within 120 sec were placed on the platform for 10 sec. Spatial memory for each rat was determined based on the probe trial. Hidden platforms in the target quadrant were removed after training for 5 days. On day 6, a probe trial was performed and the rats were allowed to swim freely in the pool for 2 min.

### Y-maze test

Spatial memory status in rats was tested by measuring the percentage of alternation in Y-maze test according to the previous description (26). The test device consisted of three equal-length arms (50×18×35 cm) in a Y-shape and an intermediate region. Rats were placed at the end of either arm, and were allowed to explore freely for 8 min. Subsequently, the total number and sequence of entries into each arm were recorded. The percentage of alternations was determined based on the following equation: (spontaneous alternations)/(total number of arm entries-2).

### Positron-emission tomography and image processing

Before euthanasia, rats fasted for 12 h were maintained under anesthesia with 1.5% isoflurane for PET. After intravenous injection of 18F-FDG (800 μCi, 29.6 MBq), the entire body of the rat was continuously scanned for 21 min with a PET scanner (Metis 1800, Madic Technology, Linyi, China) in the coronal, sagittal and transverse dimensions, followed by a focused scan of the brain for 15 min, especially the hippocampus. The brain glucose uptake was analyzed by measuring the mean standard uptake values (SUV_Mean_) using PMOD 4.1 software (PMOD Technology, Zurich, Switzerland).

### Histological analysis

Hippocampal tissues were fixed with 4% paraformaldehyde and embedded in paraffin. Paraffin sections (5 μm) were stained with hematoxylin-eosin to evaluate the structure of four vital sub-regions of the hippocampus. After dewaxing, the sections were stained with hematoxylin staining solution (G1004, ServiceBio, Wuhan, China) for 5 min, and then stained with eosin staining solution (G1001, Wuhan, China, ServiceBio, Wuhan, China) for 5 min. In addition, Nissl staining was performed on the sections to evaluate the neuronal damage. The samples were processed in the same way as H&E staining, then stained with Nissl’s staining solution (G1036, ServiceBio, Wuhan, China) for 5 min, and finally sealed with neutral resin. Digital slides were prepared by a Pannorama digital slide scanner (Pannoramic DESK, P-MIDI, P250, and P1000, 3DHISTECH, Budapest, Hungary).

### TUNEL assay

Apoptosis in the hippocampus tissues was detected by commercial TUNEL Apoptosis kit (C1086, Beyotime Biotechnology, Shanghai, China), according to the manufacturer’s instructions. The positive cells in each group were counted under a microscope. Apoptotic cells were stained in green color.

### Transmission electron microscopy

Tissue squares (1 mm×1 mm×1 mm) were fixed with electron microscopy fixative (G1102, ServiceBio, Wuhan, China) for 2-4 hrs at 4°C, and then were post-treated in 1% osmium tetroxide for 2 hrs at 4°C. Subsequently, the samples were dehydrated through an ethanol series and infiltrated using acetone and 812 embedding medium (905529-77-4, SPI). After complete polymerization, the sections were observed under the TEM (Hitachi, HT-7700, Japan).

### Immunohistochemistry

Paraffin sections (5 μm) were deparaffinized, and washed 3 times with PBS (G0002-2L, Servicebio, Wuhan, China). The antigens were retrieved in a microwave oven with citrate buffered saline (C1032, Solarbio, Beijing, China). Sections were incubated overnight with primary antibodies including p-tau (Ser404) (1:200, ab92676, Abcam, Cambridge, USA) and p-tau (Ser396) (1:4000, ab109390, Abcam, Cambridge, USA), followed by washing three times with PBS. Sections were then incubated with a universal two-step detection kit (PV-9000, ZSGB-BIO, Beijing, China) following the manufacturer’s instructions. After washing 3 times with PBS, the sections were stained with diaminobenzidine (DAB, ZLI-9017, ZSGB-BIO, Beijing, China) and hematoxylin. Finally, the sealed sections were made into digital slides by a panoramic digital slide scanner (Panorama Desk, P-MIDI, P250 and P1000, 3DHISTECH).

### Western blot analysis

Hippocampal tissues were homogenized in RIPA cold buffer (89901; Thermofisher, USA) containing protease inhibitor (ST506, Beyotime Biotech, Shanghai, China). Protein samples were quantified using the BCA protein assay kit (E-BC-K318-M, Elabscience, Wuhan, China). Protein samples (50 μg) were separated by SDS-PAGE gel (PG212, EpiZyme, Shanghai, China) and then transferred to a polyvinylidene fluoride (PVDF) membrane. PVDF membranes were blocked with 5% nonfat dry milk for 1 h, and incubated with primary antibody overnight at 4°C [p-tau (Ser404), 1:2000, ab92676, Abcam, USA; p-tau(Ser396), 1:50000, ab109390, Abcam, USA; Tau, 1:10000, sc-32274, Santa Cruz Biotechnology, Beijing; p-PI3K, 1:1000, 13857S, Cell Signaling Technology, USA; PI3K, 1:1000, 3358S, Cell Signaling Technology, USA; Akt, 1: 1000, 4685S, Cell Signaling Technology, USA; p-Akt, 1:2000, 4060S, Cell Signaling Technology; GSK3β, 1:1000, 9315S, Cell Signaling Technology, USA; p-GSK3β, 1:1000, 9315S, Cell Signaling Technology, USA; Bcl-2, 1:5000, 60178-1-Ig, Proteintech, China; Bax, 1:10000, 50599-1-Ig, Proteintech, China; Caspase 3, 1:1000, 9662S, Cell Signaling Technology, USA; cleaved Caspase 3, 1:1000, 9664S, Cell Signaling Technology, USA; β actin, 1:20000, 66009-1-Ig, Proteintech, China]. Then, the membrane was washed and incubated with secondary antibodies (goat anti-mouse IgG, 1:10000, ab216776, Abcam; goat anti-rabbit IgG, 1:10000, ab6721, Abcam). Protein bands were visualized by ECL (Millipore) and quantified using ImageJ software (National Institutes of Health).

### Statistical analysis

Data were analyzed using Graph Pad Prism 8.0 (San Diego, CA, USA). Data were presented as mean ± standard error of mean. One-way ANOVA was utilized to compare the differences between groups, together with Tukey’s multiple comparison test. Statistical outliers were determined using the Grubbs test. *P <*0.05 was considered to be statistically significant.

## Results

### SG improved basic metabolic parameters in diabetic rats

**Figure 2:**
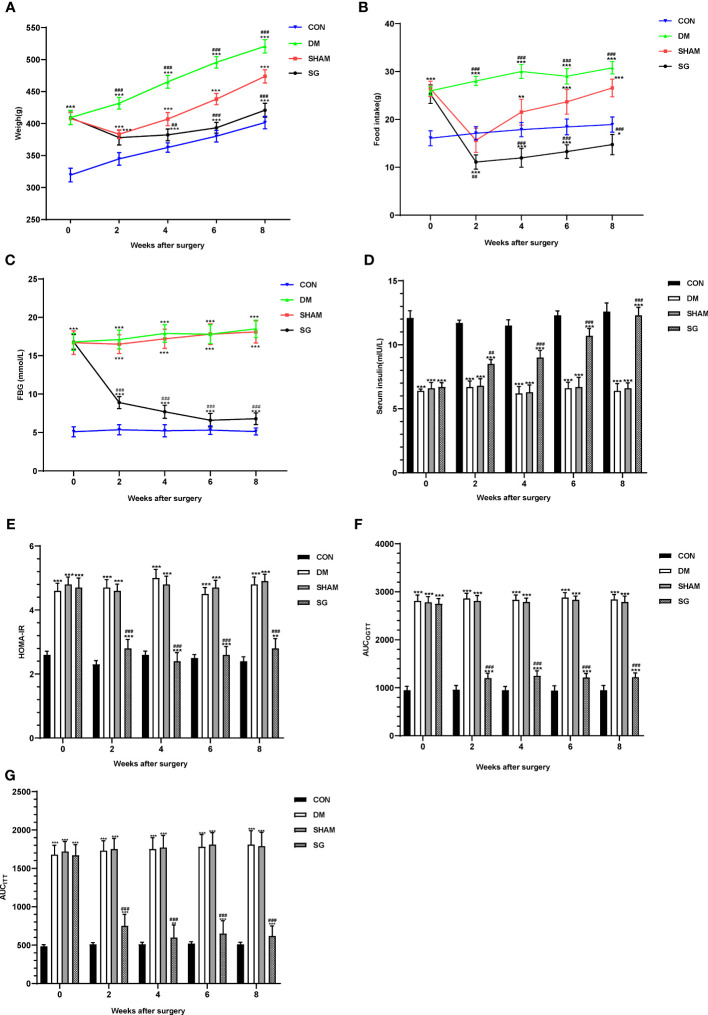
Changes of metabolic parameters including body weight **(A)**, food intake **(B)**, FBG **(C)**, serum insulin **(D)**, HOMA-IR **(E)**, AUC_OGTT_ **(F)**, and AUC_ITT_ **(G)** before and after surgery. Data were expressed as means ± SEM for n = 10 per group. ^**^ *p* < 0.01 vs. CON group, ^***^ *p* < 0.001 vs. CON group; ^##^ *p* < 0.01 vs. SHAM group, ^###^ *p* < 0.001 vs. SHAM group. FBG, fasting blood glucose; HOMA-IR, homeostasis model assessment of insulin resistance; AUCOGTT, the area under the curve of the oral glucose tolerance test; AUCITT, the area under the curve of the insulin tolerance test; CON, control; DM, diabetes mellitus; SHAM, sham operation; SG, sleeve gastrectomy.

The body weight and food intake were significantly higher in the DM group than these of the CON group. Compared with the DM group, significant decrease was noticed in the body weight and food intake of rats in SG group (**Figures 2A, B**). In addition, compared with the SHAM group, the FBG showed significant decrease in the SG group within 2 weeks after surgery (**Figure 2C**). By recording FBG and serum insulin levels for HOMA-IR assessment, we found a significant decrease in insulin resistance in the SG group compared to the sham group (**Figures 2C-2E**). Consistently, AUC_OGTT_ and AUC_ITT_ further validated the improvement in insulin resistance in the SG group (**Figures 2F, G**). All these indicated that SG could significantly improve the basic metabolic parameters in diabetic obese rats.

### SG ameliorated cognitive function in diabetic rats

**Figure 3:**
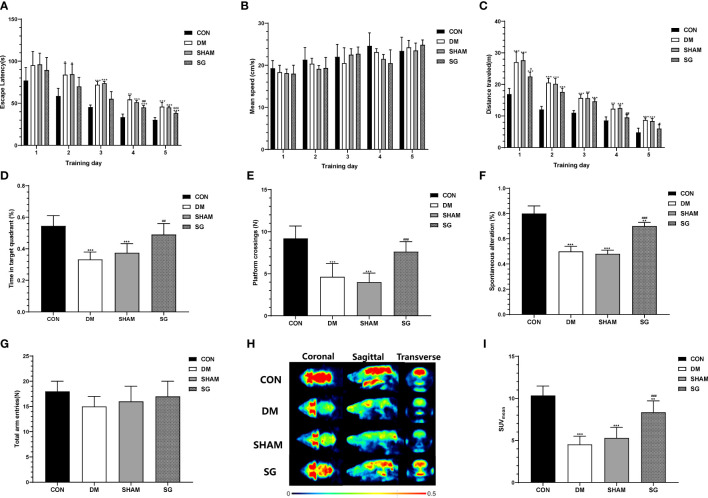
SG induced significant improvement in the animal behaviors in the Morris water maze test **(A-E)** and Y-maze test **(F, G)**, and PET imaging system validated the improvement of glucose uptake in brain **(H, I)**. Data were expressed as means ± SEM for n = 10 per group. ^*^ *p* < 0.05 vs. CON group, ^**^ *p* < 0.01 vs. CON group, ^***^ *p* < 0.001 vs. CON group; ^#^ *p* < 0.05 vs. SHAM group, ^##^ *p* < 0.01 vs. SHAM group, ^###^ *p* < 0.001 vs. SHAM group. PET, positron-emission tomography; SUV_Mean_, the average standard uptake value; CON, control; DM, diabetes mellitus; SHAM, sham operation; SG, sleeve gastrectomy.

The escape latency of the rats in the DM group and the SHAM group was significantly longer than that of the control (**Figure 3A**). The escape latency in the SG group was significantly shorter than that of the DM group (**Figure 3A**). Meanwhile, there was no statistical difference in the swimming speed of the rats in each group (**Figure 3B**). The travelled distance of rats in each group showed gradual decrease in a time-dependent manner. The travelled distance in the SG group was significantly shorter than the SHAM group on day 4 and 5 (**Figure 3C**). Compared with normal rats, the percentage of time in target quadrant (**Figure 3D**) and the number of platform crossing (**Figure 3E**) were significantly shortened in the DM and SHAM groups. In contrast, the percentage of time and number of platform crossings in SG group showed significant increase compared with the SHAM group (**Figures 3D, E**). These indicated that SG significantly improved spatial memory and learning ability in diabetic rats. In addition, the Y-maze test showed that the percentage of spontaneous alternation in the DM group was lower than that in the control group, while the percentage of spontaneous alternation in the SG group was significantly different compared to the SHAM group (**Figure 3F**), with no significant changes in total arm entries (**Figure 3G**). This suggested that SG may improve the ability of diabetic rats to recognize novel environment, and could partially improve the DCD.

### SG significantly improved cerebral glucose uptake in diabetic rats

We evaluated glucose uptake in brain tissues using a PET scanner (**Figure 3H**). The SUV_mean_ of the control group and SG group was significantly higher than that of the DM group and sham group, respectively (**Figure 3I**). These results suggested that SG improved the diabetes-induced obstruction of cerebral glucose uptake.

### SG significantly reversed hippocampal histopathology in diabetic rats

**Figure 4:**
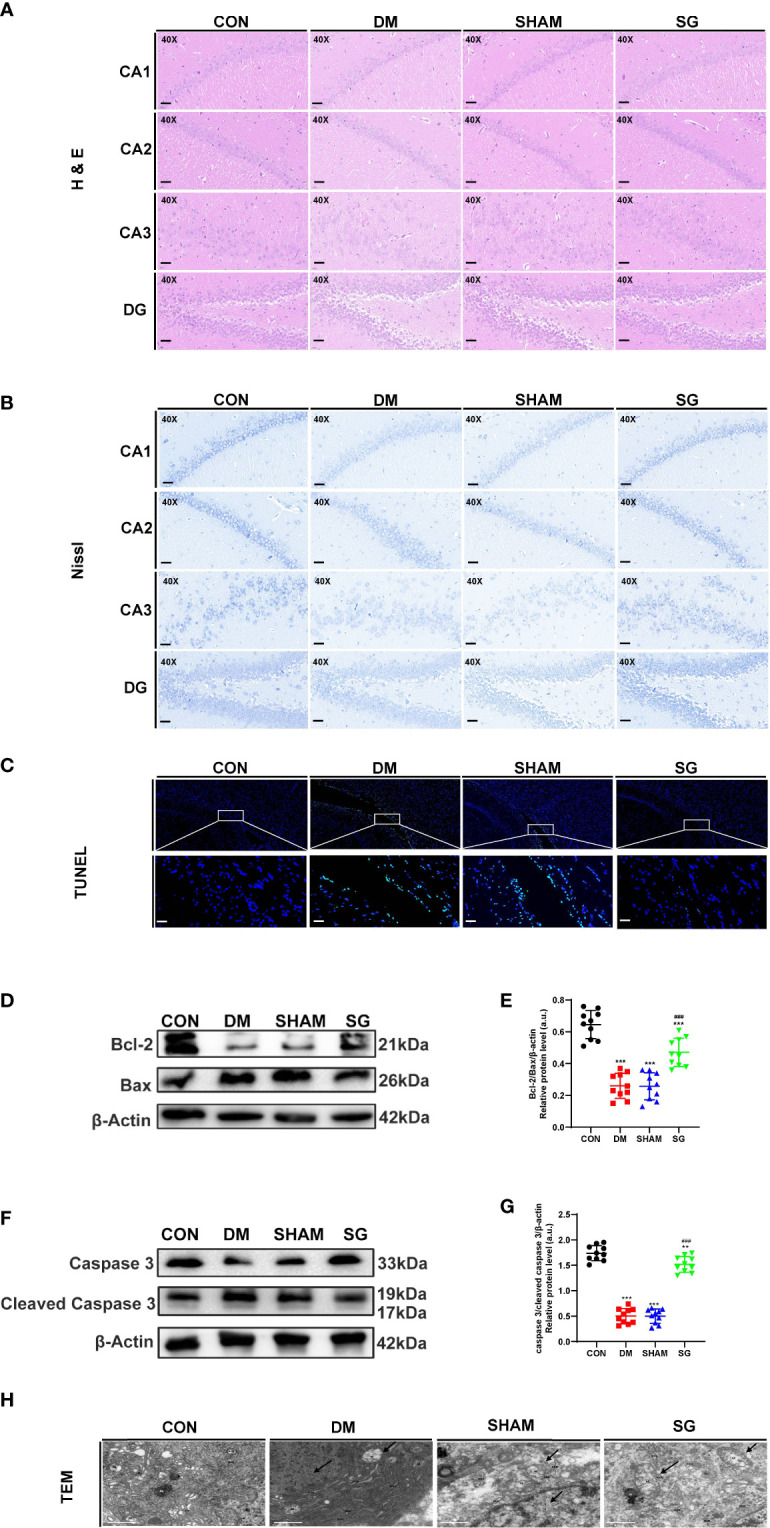
SG reversed the histological features **(A)**, induce the increase of Nissl’s bodies **(B)**, and inhibited the apoptosis of hippocampus neurons **(C-G)**, and improved the microstructure of hippocampus neurons under TEM **(H)**. The scale bar for A-C was 100 µm, while that for H was 1 µm. Data were expressed as means ± SEM for n = 10 per group. ^**^ *p* < 0.01 vs. CON group, ^***^ *p* < 0.001 vs. CON group; ^###^ *p* < 0.001 vs. SHAM group. H&E, hematoxylin and eosin; TUNEL, terminal deoxynucleotidyl transferase-mediated dUTP nick end labeling; CA1, cornu ammonis1; CA2, cornu ammonis2; CA3, cornu ammonis3; DG, dentate gyrus; M, mitochondrion; N, nucleus; RER, rough endoplasmic reticulum; GO, Golgi apparatus; CON, control; DM, diabetes mellitus; SHAM, sham operation; SG, sleeve gastrectomy.

Changes in the hippocampus tissue underlie cognitive decline as central nervous system diseases and diabetes progress (27, 28). Compared with the CON group, the number of neurons in the hippocampal cornu ammonis (CA)1, CA2, CA3, and dentate gyrus (DG) regions of the DM group showed significant decrease, which were featured by presence of swollen cells, nuclear fragmented or disappearance, and irregular arrangement of cell. Notably, SG significantly improved these changes (**Figure 4A**). To assess the damage of hippocampal neurons, the hippocampus of each group was analyzed using Nissl staining. DM resulted in the reduction of Nissl bodies in the CA1, CA3 and DG regions of the hippocampus, indicating significant neuronal damage. Whereas, the number of Nissl bodies in the SG group showed significant increase compared with SHAM group (**Figure 4B**). In conclusion, SG ameliorated the histological changes in the hippocampus induced by diabetes.

### SG alleviated hippocampal neuronal apoptosis in diabetic rats

In DM group, there was a significant increase in the number of positive neurons in the hippocampus, indicating significant increase in the apoptosis compared with the CON group. In contrast, SG alleviated the situation of neuronal apoptosis (**Figure 4C**). Western blot analysis showed that Bcl-2 and Caspase 3 were down-regulated in DM group and SHAM group compared with these of the CON group, while significant up-regulation was seen in the expressions of Bax and cleaved Caspase 3 in DM group (**Figures 4D, F**). The ratio of Bcl-2 to Bax and Caspase 3 to cleaved Caspase 3 in the SG group showed significant increase compared with that of SHAM group (**Figures 4E, G**). Taken together, we concluded that SG can ameliorate diabetes-induced apoptosis of hippocampal neurons.

### SG significantly improved the fine structure of hippocampal neurons

TEM indicated pyknosis, severe edema, condensed cell matrix, obvious swelling of organelles in the hippocampal neurons in the DM group and SHAM group (**Figure 4H**), together with obvious vacuolar degeneration. In CON group and the SG group, the nuclear membrane was intact. In addition, the chromatin was uniform, and the cell membrane was intact. Moreover, the intracellular matrix was abundant and evenly distributed. It was worth noting that the mitochondria in the SG group were slightly swollen, and the cristae were fragmented and reduced, but not as severe as the SHAM group.

### SG inhibited tau phosphorylation in the hippocampus of diabetic rats

**Figure 5:**
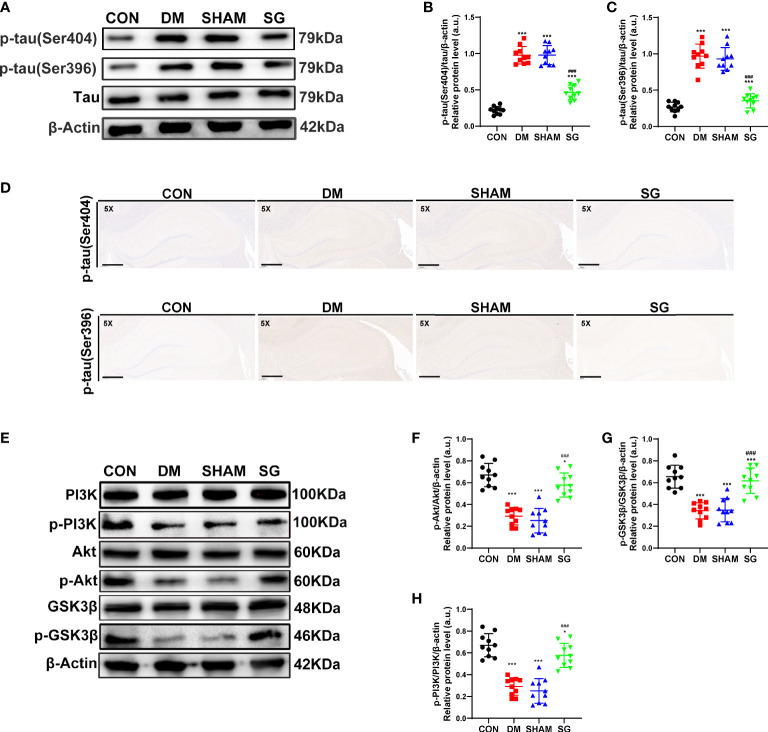
SG inhibited the phosphorylation of tau **(A-D)** and activated the PI3K signaling pathway **(E-H)**. The scale bar for D was 800 µm. Data were expressed as means ± SEM for each group (n = 10). ^*^ *p* < 0.05 vs. CON group, ^***^ *p* < 0.001 vs. CON group; ^###^ *p* < 0.001 vs. SHAM group. CON, control; DM, diabetes mellitus; SHAM, sham operation; SG, sleeve gastrectomy.

There was increased phosphorylation of tau at Ser404 and Ser396 in the hippocampus of DM and SHAM group compared with CON group (**Figure 5A**). In contrast, SG decreased the phosphorylation of these two tau sites (**Figures 5B, C**), which was verified by IHC results (**Figure 5D**). Thus, SG alleviated hyperphosphorylation of tau in the hippocampus of diabetic rats.

### Effects of SG on PI3K signaling pathways

Compared with the CON group, the hippocampal expression of p-PI3K, p-Akt, and p-GSK3β showed significant decrease in both DM and SHAM groups, while SG induced significant increase in their expression (**Figure 5E**). The ratios of p-PI3K to total PI3K, p-Akt to total Akt and p-GSK3β to total GSK3β were consistent with the above results (**Figures 5F-5H**). In conclusion, we infer that SG partially ameliorated diabetes-induced cognitive impairment, which was associated with activation of the PI3K signaling pathway.

## Discussion

DM and its complications were indeed a serious threat to global health. On this basis, there has been widespread interests in the treatment of diabetes (1). DCD has been more and more widely recognized as a serious complication for DM (29). Slowly progressive DCD occurs in all age groups, not limited to the aged population (30). The main pathological features of DCD include hyperphosphorylation of tau and apoptosis of hippocampal neurons, leading to progressive impairment of hippocampal function (31–33). A large number of DCD patients are more likely to present mild cognitive impairment (MCI) or dementia, showing a poor prognosis even after treatment (33, 34). Conventional treatment options for DCD include measurements for lowering blood glucose, lifestyle interventions, and cognitive rehabilitation training. In addition, delivery of insulin to targeted brain tissues has been proposed as a potential strategy for treating cognitive impairment in DM patients (35). Unfortunately, this strategy is not universally effective due to poor adherence and individual variability (36). Therefore, it is urgently to develop more effective treatment options for these patients.

In the 1990s, bariatric surgery began to be recognized as a form of inducing weight loss, which may improve the symptoms of DM and its complications (37). Recently, it has gradually considered as the best treatment strategy for DM and obesity, with the advances in the surgical safety (38). In terms of animal models, due to the similarity of metabolic characteristics, the most commonly used diabetes model is the DM rodent model, which is used to investigate the pathogenesis and treatment of DM (39). In this study, such model was used to investigate the therapeutic effects of SG on DCD and its associated mechanisms. According to the previous studies, cognitive decline was sufficiently induced in this model about 8-9 weeks after induction of DM (40, 41). Our data showed that the cognitive function of rats was significantly impaired at week 12 after DM. Meanwhile, SG showed significant effects on reducing body weight, together with improving hyperglycemia and reversing insulin resistance. However, some rats showed signs of rebound in body weight and blood sugar after surgery, which we consider to be caused by maintaining a high-fat diet during the experiments. The CA1, CA3 and DG regions of the hippocampus were crucial for learning and spatial memory, and there was a unidirectional tri-synaptic pathway between these regions (42). In this study, we found that cognitive function decreased. HE and Nissl staining revealed neuronal damage in the CA1, CA3, and DG regions in DM rats. Interestingly, these negative effects were improved to varying degrees after SG. The above notion was further supported by the changes in neuronal microstructure under TEM. Taken together, SG could improve the symptoms in diabetic rats with DCD.

PET, employing molecules labeled with positron-emitting radioisotopes to provide direct and specific measurements of biochemical processes in regions of interest, has been used to gain a deeper understanding on the neural mechanisms underlying behavioral and cognitive processes (43). In cognition-related regions, there is an association between insulin resistance and reduced brain glucose metabolism (44). Therefore, PET scans of rat brains were performed and SUV was utilized to assess glucose uptake in brain, particularly the hippocampus. PET scan showed significant increase in the SUV in SG group, but it did not reach the level of the CON group. And confirmed that SG significantly reversed cerebral glucose uptake in diabetic rats.

Pathological alternations of hippocampus are closely associated with the pathogenesis of DCD with the main pathological manifestations as hyperphosphorylation of tau and increased neuronal apoptosis (45). These pathological changes would trigger the inhibition of PI3K/Akt signaling pathway (46). Previous study indicated that many Tau sites can be phosphorylated and inactivated, including Ser404, Ser396, Ser202 and Thr205 (47). In our study, Western blot analysis and IHC showed that the expression of p-tau (Ser404) and p-tau (Ser396) showed significant up-regulation in the DM group and SHAM group compared with those of the CON group. According to the previous studies, DM could mediate the increased apoptosis of hippocampal neurons by inhibiting the PI3K pathway (48, 49). Consistently, our TUNEL assay indicated the increased expression of apoptosis-related proteins in hippocampus. Therefore, we concluded that SG could improve the pathological changes. Additionally, our data provided solid evidence for the neuroprotective effects of bariatric surgery by inhibiting neuronal apoptosis and tau phosphorylation.

**Figure 6:**
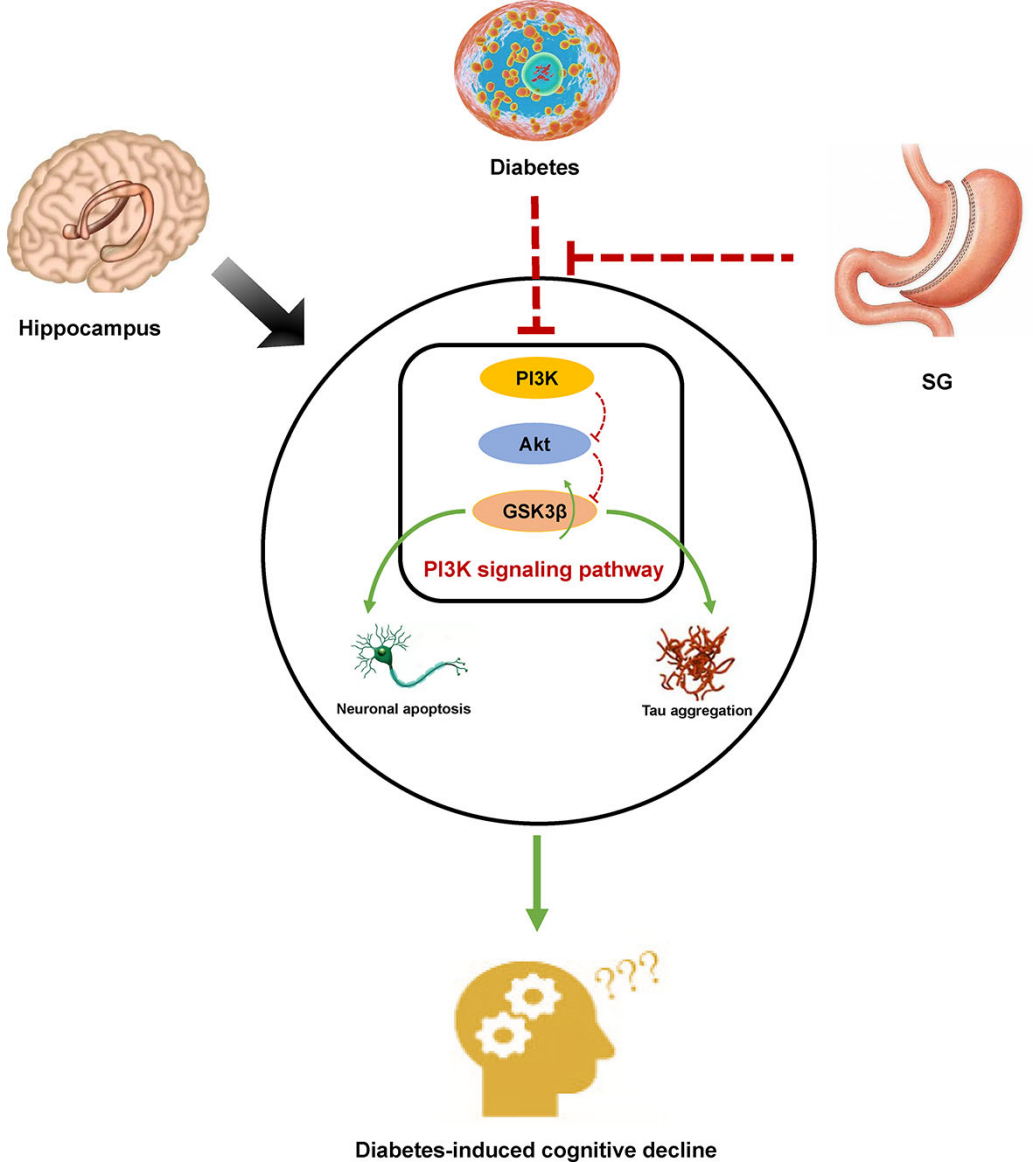
Diagram of a possible mechanism by which SG improved DCD. In DM rats, inhibition of PI3K signaling resulted in histological changes in the hippocampus by promoting tau phosphorylation and inducing apoptosis of hippocampal neurons. Activation of PI3K signaling pathway was associated with SG-induced amelioration of DCD.

PI3K signaling pathway plays crucial roles in several biological processes, such as glucose homeostasis, cell growth and proliferation (50). The activation of Akt and inactivation of GSK3β was highly depending on phosphorylation of corresponding serine residues, which functioned as serine/threonine kinases (51, 52). PI3K was activated by direct interaction with insulin receptor substrate 1 (IRS-1), and then the Akt was phosphorylated, which in turn induced the phosphorylation of GSK3β and ultimately promoted the balance of blood glucose (53). It has been assumed that there is a potential link between PI3K signaling pathway and the pathogenesis of DM or Alzheimer’s disease (AD) as there is confirmed impairment of PI3K signaling pathway in the DM. Inhibition of glycogen synthesis and inactivation of tau have been reported to trigger hyperglycemia and cognitive decline, respectively (54). Meanwhile, increased ratio of GSK3β to p-GSK3β resulted in increased apoptosis of hippocampal neurons in DM rats (55). Notably, the expression of p-PI3K, p-Akt, and p-GSK3β was significantly up-regulated after SG, which suggested that the activation of PI3K signaling pathway may play an important role in the attenuation of DCD mediated by SG (**Figure 6**).

Indeed, our study has some limitations. Some patients may present rebounding of blood glucose and weight even after bariatric surgery (56). It is still difficult to predict the state of diabetes-induced cognitive impairment following re-elevation of blood glucose and weight. Therefore, it is necessary to extend the observation time in the following studies to discuss the long-term effects of SG on diabetes-induced cognitive impairment. In addition, both glycemic control and weight loss have positive effects on metabolic status. Thus, it is not clear which is the crucial factor in the improvement of cognitive decline in SG-induced diabetes. On this basis, further studies are required to illustrate the exact mechanism of diabetes-induced cognitive impairment improvement. Finally, to further elucidate the mechanism by which SG attenuated the diabetes-induced cognitive impairment, we need to explore the interaction between GSK3β, Tau and neuronal apoptosis.

In conclusion, SG could reverse the tissue morphology of the hippocampus, decrease of glucose uptake in the hippocampus, and attenuate cognitive dysfunction induced by hippocampal neuronal apoptosis and hyperphosphorylation of Tau in DM rats. Additionally, the SG could reverse the inhibition of PI3K signaling pathway in rats with DCD. The reduction of diabetes-induced cognitive function by SG was associated with reactivation of the PI3K signaling pathway. In the future, inhibition of PI3K signaling may be a potential target for treating patients with DCD.

## Data availability statement

The raw data supporting the conclusions of this article will be made available by the authors, without undue reservation.

## Ethics statement

The animal study was reviewed and approved by Institutional Animal Care and Use Committee of Shandong Qianfoshan Hospital, Shandong University.

## Author contributions

GZ and MZ contributed to conception and design of the study. Material preparation and data collection were performed by HD, CL, SZ, BL, QX and BS. Data analysis was performed by SL, SD, XM and YZ. HD, CL and SZ wrote the first draft of the manuscript. BL, QX, BS, SL, SD, XM and YZ wrote sections of the manuscript. GZ and MZ provided critical review of the article. All authors contributed to manuscript revision, read, and approved the submitted version.

## Funding

This project was supported by the National Natural Science Foundation of China (Grant No. 81873647) and Major Basic Research Project of Natural Science Foundation of Shandong Province (Grant No. ZR2020ZD15).

## Conflict of interest

The authors declare that the research was conducted in the absence of any commercial or financial relationships that could be construed as a potential conflict of interest.

## Publisher’s note

All claims expressed in this article are solely those of the authors and do not necessarily represent those of their affiliated organizations, or those of the publisher, the editors and the reviewers. Any product that may be evaluated in this article, or claim that may be made by its manufacturer, is not guaranteed or endorsed by the publisher.

## Supplementary material

Supplementary Figure 1 DM induced significant impairment in the animal behaviors in the Y-maze test(A, B). Data were expressed as means ± SEM for n = 10 per group. ^*^ *p* < 0.05 vs. CON group, ^**^ *p* < 0.01 vs. CON group, ^***^ *p* < 0.001 vs. CON group. CON, control; DM, diabetes mellitus; SHAM, sham operation; SG, sleeve gastrectomy.
