# Supplementary figures and images for: Sleeve gastrectomy attenuated diabetes-related cognitive decline in diabetic rats
Source: Front Endocrinol (Lausanne). 2022 Nov 3;13:1015819. doi: 10.3389/fendo.2022.1015819 (PMC9669300; doi:10.3389/fendo.2022.1015819)

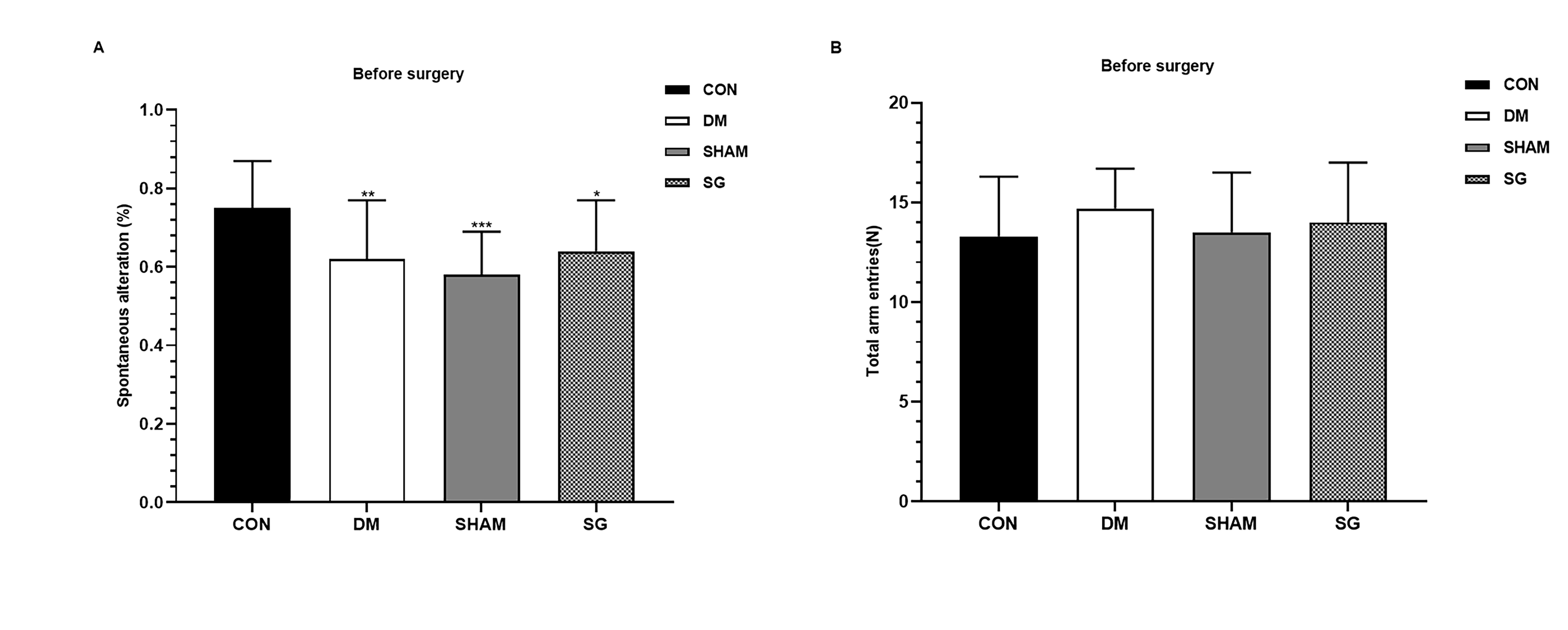

Supplement: Supplementary Figure 1 — DM induced significant impairment in the animal behaviors in the Y-maze test(A, B). Data were expressed as means ± SEM for n = 10 per group. * p < 0.05 vs. CON group, ** p < 0.01 vs. CON group, *** p < 0.001 vs. CON group. CON, control; DM, diabetes mellitus; SHAM, sham operation; SG, sleeve gastrectomy. [file Image_1.tif]
